# Supplementary material for: PRFect: a tool to predict programmed ribosomal frameshifts in prokaryotic and viral genomes
Source: BMC Bioinformatics. 2024 Feb 22;25:82. doi: 10.1186/s12859-024-05701-0 (PMC10885494; doi:10.1186/s12859-024-05701-0)
Supplement: Supplementary file 1 — Additional file 1. The ten different motifs that PRFect uses as slippery sites to then predict which of those 3 sites find are involved in programmed ribosomal frameshifting. The linear realtionship between the MFE and the GC% content. The full results of PRFect, FSFinder2, and KnotInFrame. The nucleotide frequency at each base location in the ten motifs. [file 12859_2024_5701_MOESM1_ESM.pdf]

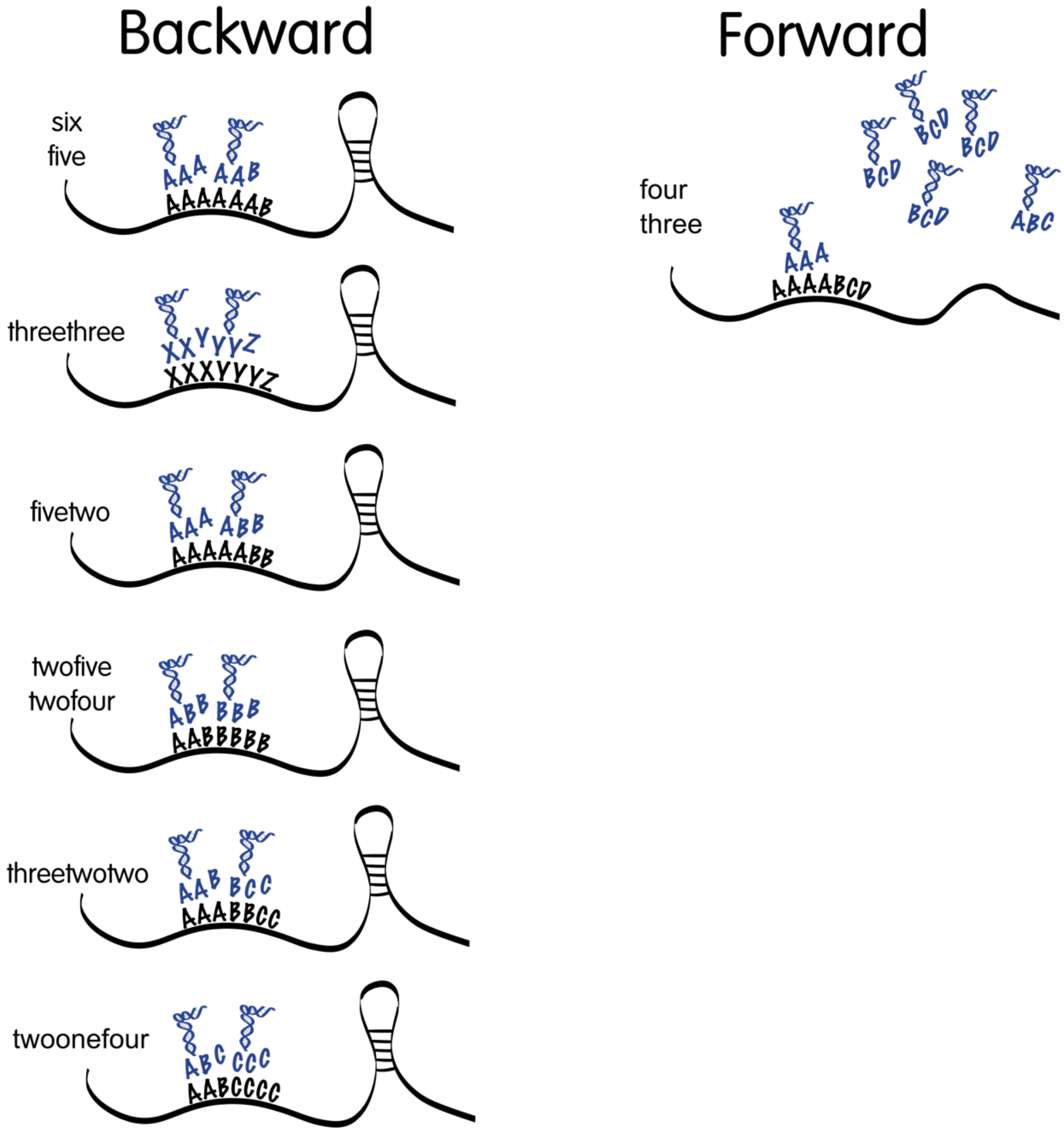

Supplementary Figure 1) The ten different motifs that PRFect uses as slippery sites to then predict which of those sites find are involved in programmed ribosomal frameshifting. Shown is the originally proposed XXXYYYZ motif for backward frameshifting, along with 9 other proposed motifs that might also allow for the wobble base pairing mismatch initialy discovered with the XXXYYYZ motif. The motifs are named according to the repeated bases (i.e. six is 6 bases in a row), are shown in descending order based on the probabilty of seeing that motif by chance alone, and some are grouped together for brevity. The black letter correspond to the bases (codons) of the mRNA transcript while the blue base correspond to the bases (anti-codons) of the two bound tRNA. The tRNA are shown in their frameshifted position (backwards or forwards) and show how the third weak wobble position of the tRNA might not match the new frameshifted pairing. For example in the original threethree motif the tRNAs XXY and YYZ are bound to the mRNA and then shift back one base so that only their 1st an d 2nd bases are paired correctly while the 3rd wobble bases are mismatched. The probabilty of seeing three (and four) bases in a row in given genome is quite common, so for the forward frameshifts we also require that the codon of the waiting A-site (ABC) is rarer than the codon of the +1 A-site (BCD). The codon rarities are calcualted at run time on the input genome by iterating through all of its coding genes and counting the occurrence of each of the 64 different codon posibilties.

Requiring the +1 A-site to be more common than the waiting +0 A-site codon in forward frameshifts helps to cut down on false-positive preditions but mainly serves to speed up runtimes; since three bases in a row is quite common and calculting the MFE of a window is computational time intensive.

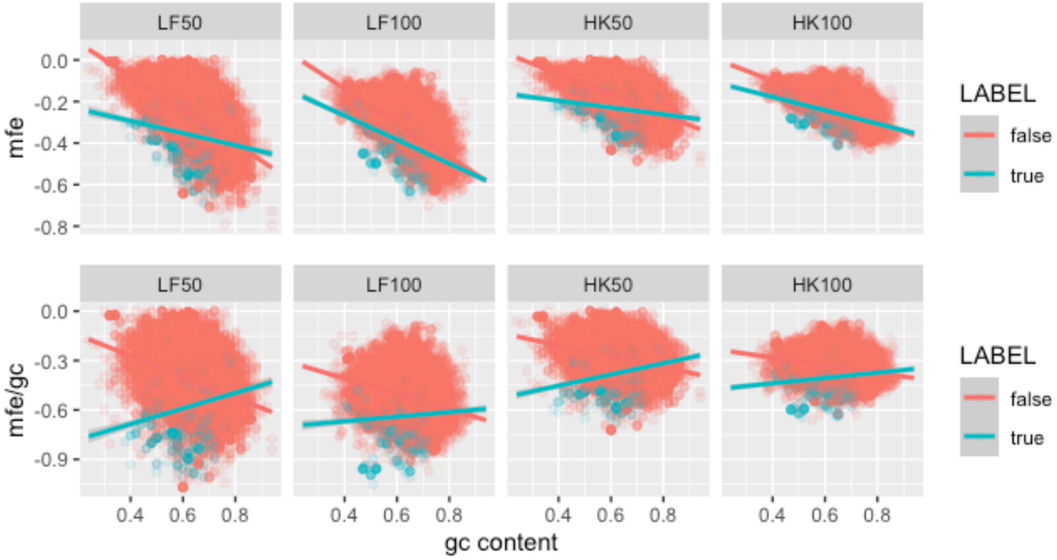

Supplementary Figure 2) The programs LinearFold (LF) and HotKnots (HK) were used to calculate the minimum free energy (MFE) for 50bp and 100bp windows downstream of slippery sites in the SEAPHAGES data from *joined* genes (*true*) known to frameshift and from genes that are adjacent and not *joined* in the annotation file (*false*). The top show the linear realtionship between the MFE and the GC% content thaat would impair a model trained on data biased towards high or low GC content genomes. The genomes of the SEAPHAGES data tend to be higher in GC content, so in order to help PRFect perform on genomes of any GC% content, we divide the MFE by the GC to help normalize the MFE to the GC.

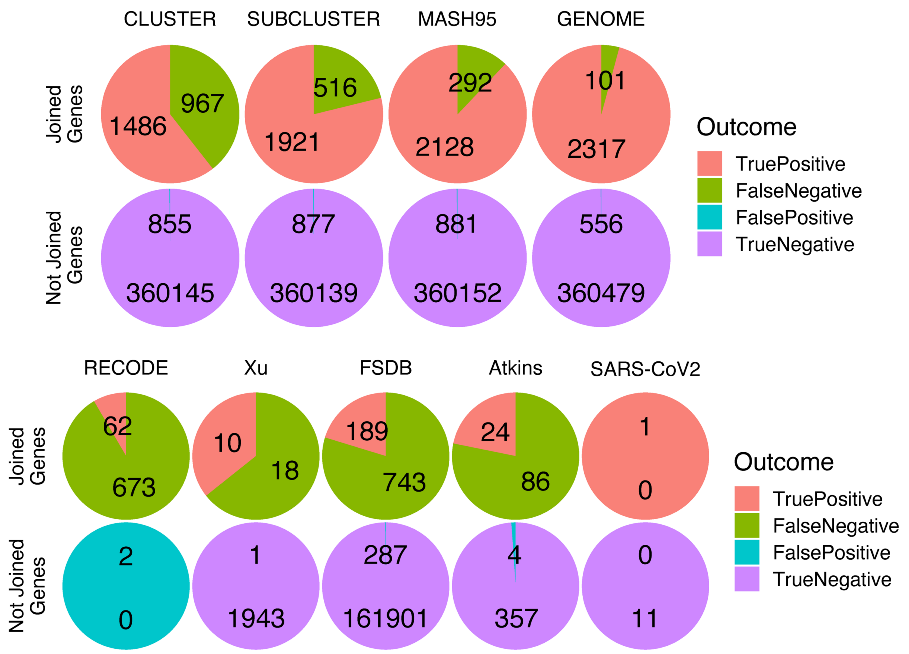

Supplementary Figure 3) The full results of PRFect on the various datasets

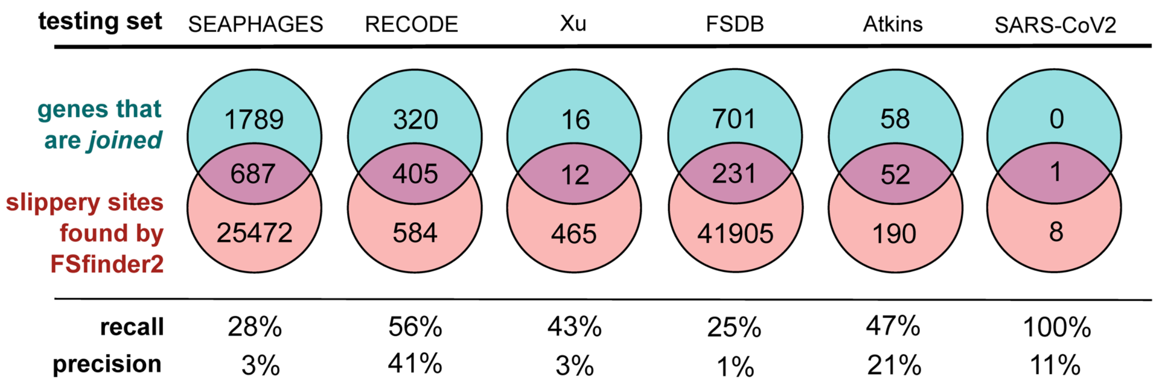

Supplementary Figure 4) The performance of FSFinder2 on the various datasets.

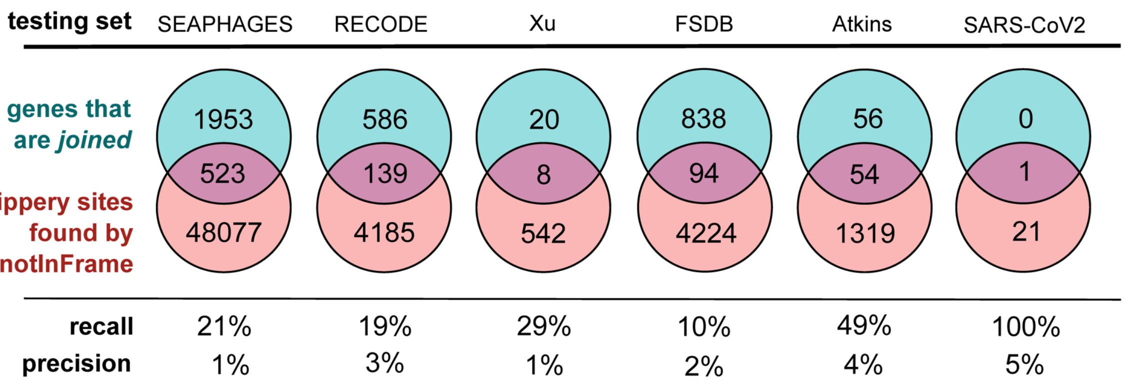

Supplementary Figure 5) The performance of KnotInFrame on the various datasets.

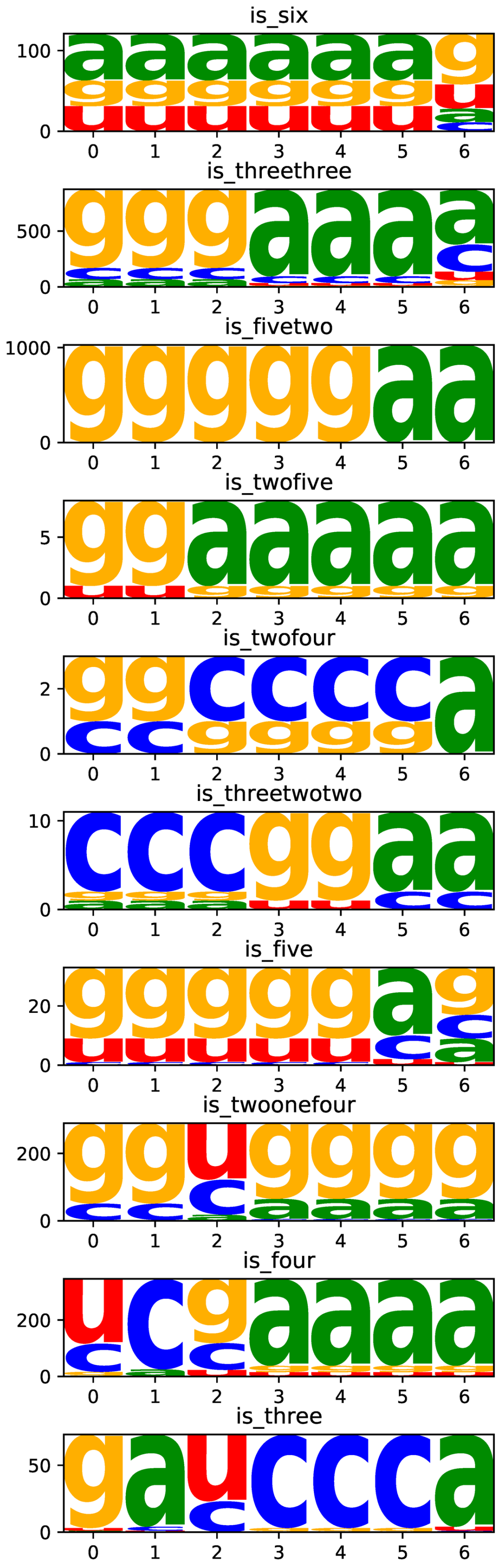

Supplementary Figure 6) The nucleotide frequency at each base location in the various motifs of the true-positive slippery sites
